# Saliva as a Cure for Rheumatism

**Published:** 1873-10

**Authors:** 


### SALIVA AS A CURE FOR RHEUMATISM.

Some one writes to the editor of the *Medical Press and Circular* that he has repeatedly cured himself of rheumatism by rubbing the affected part with his own saliva. As saliva is too common to become an article of traffic, the new treatment is not likely to become popular.—*Med. & Sur. Reporter.*

